# Supplementary figures and images for: Amoxicillin/clavulanate in combination with rifampicin/clarithromycin is bactericidal against Mycobacterium ulcerans
Source: PLoS Negl Trop Dis. 2024 Apr 4;18(4):e0011867. doi: 10.1371/journal.pntd.0011867 (PMC10994486; doi:10.1371/journal.pntd.0011867)

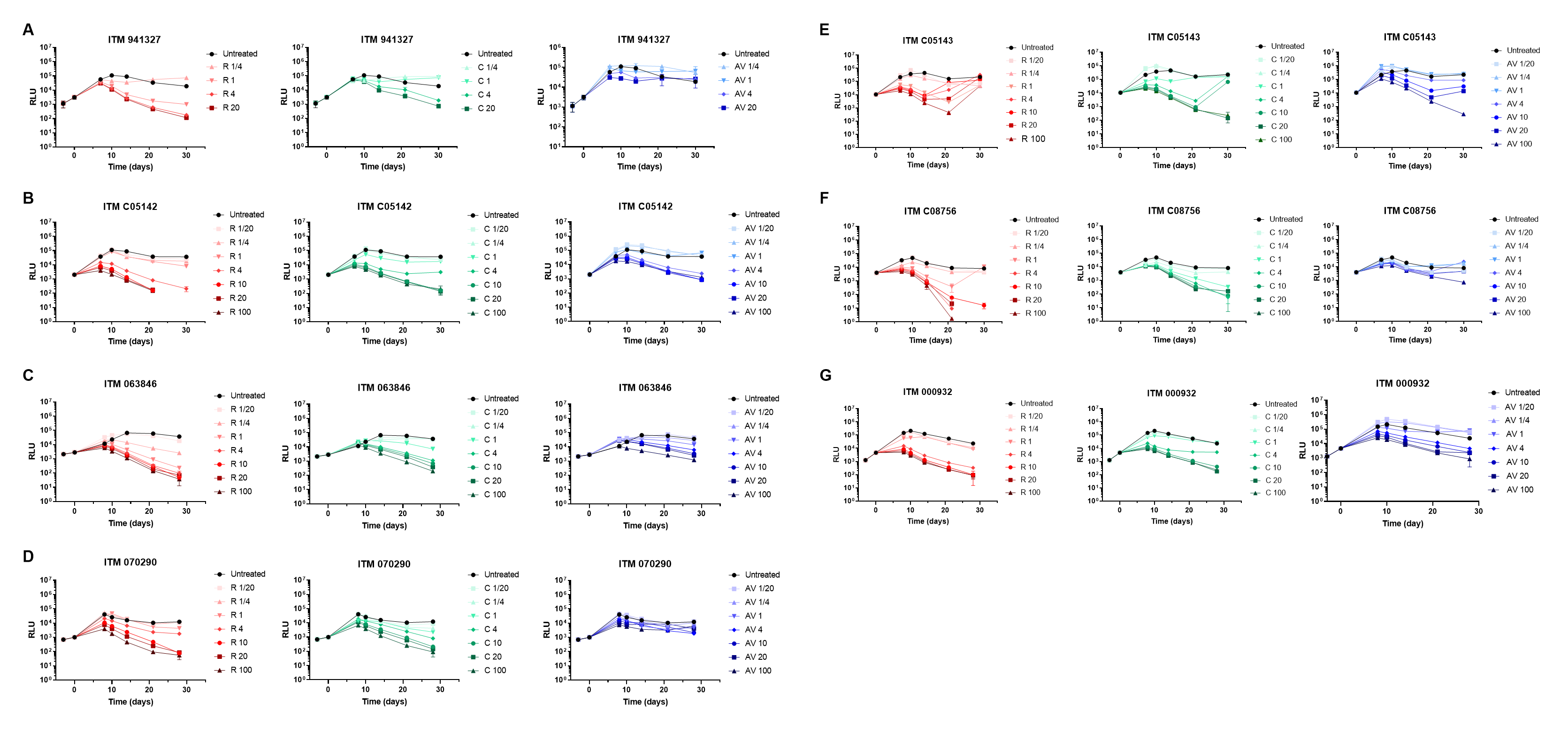

Supplement: S1 Fig — (A) ITM 941327, (B) ITM C05142, (C) ITM 063846, (D) ITM 070290, (E) ITM C05143, (F) ITM C08756, (G) ITM 000932. RLU, Relative Light Units; R, rifampicin; C, clarithromycin; AV, amoxicillin/clavulanate. (TIF) [file pntd.0011867.s003.tif]
