# Supplementary material for: Amoxicillin/clavulanate in combination with rifampicin/clarithromycin is bactericidal against Mycobacterium ulcerans
Source: PLoS Negl Trop Dis. 2024 Apr 4;18(4):e0011867. doi: 10.1371/journal.pntd.0011867 (PMC10994486; doi:10.1371/journal.pntd.0011867)
Supplement: Attachment — Submitted filename: [Reponse_Letter] Saez-Lopez et al_BU TKA_PLOS NTDs.pdf [file pntd.0011867.s004.pdf]

Universidad Zaragoza 1542

Paul J. Converse
Academic Editor
PLOS Neglected Tropical Diseases

Stuart Blacksell
Section Editor
PLOS Neglected Tropical Diseases

Zaragoza, March 06th, 2024

Dear Editors,

We acknowledge the provisional approval subject to modifications. We are pleased to submit for publication in PLOS Neglected Tropical Diseases the revised manuscript (PNTD-D-23-01593) entitled "**Amoxicillin/clavulanate in combination with rifampicin/clarithromycin is bactericidal against *Mycobacterium ulcerans***" according to the suggestions of three reviewers.

Response to reviewers is provided in a list of changes after this letter. All authors have contributed to the work, have read and approved the manuscript, and report no conflict of interests. The authors also declare that there are no restrictions on sharing of the underlying data and/or materials.

Sincerely yours,

SAEZ LOPEZ EMMA - 71288242H
Firmado digitalmente por SAEZ LOPEZ EMMA - 71288242H
Fecha: 2024.03.06 11:23:50 +01'00'

Emma Sáez López, PhD (on behalf of all contributing authors)

University of Zaragoza

E-mail: esaez@unizar.es

Reviewers' comments:

Methods

Reviewer #1: The objectives are clear and the methods are well both described and suitable for the purposes of the study.

Reviewer #2: Based on MIC determination and checkerboard assays it has been suggested to include amoxicillin/clavulanate (AMX/CLV) in the recommended BU chemotherapy with rifampicin and clarithromycin. These data served as in vitro microbiological support for the clinical testing of the triple RIF+CLA+AMX/CLV combination treatment. In the present report, the authors have now compared different time-kill assay readouts (CFU enumeration, luminescence relative light unit determination based on ATP quantification, optical density measurements, IS2404 qPCR and 16S rRNA RT-qPCR) to capture the effect of AMX/CLV. The RIF+AMX/CLV and RIF+CLA+AMX/CLV combinations showed the strongest bactericidal activity against a standard strain (ITM 000932) with a CFU reduction at Day 28 of about three Log10. Based on the data obtained with isolate 000932, the authors used the luminiscence assay to analyze the effect of the RIF+CLA+AMX/CLV combination on a set of M. ulcerans clinical isolates at 1/4xMIC and 1xMIC concentrations of the compounds. The objectives of the study clearly articulated, and the study design is appropriate.

Reviewer #3: This is a technical laboratory study that sets out to evaluate different methods for in vitro drug susceptibility estimation with a particular eye on the difficulties of doing this with slowly growing bacteria such as M. ulcerans. The study design is reasonable in this regard.

Comments. We appreciate reviewers' comments and, based on them, have nothing to add to this section.

Results

Reviewer #1: The figures are clear and well presented but the results are sometimes difficult to understand because the authors use set concentrations which they refer to as the MIC, but these rarely correspond to the actual MIC of the different isolates. Thus, for example, in Table S2, the actual MICs are compared to the MIC as used in the assay, but

both are referred to as just MIC in the headings. This is discussed at length in the text but is still confusing to read.

Changes 1.1. The text was rewritten for more clarification.

Pages 8-9, Lines 161, 170-175. Concentrations used in the experiments were included in the methods section.

Page 9, Lines 162-167. The explanation on the calculation of the fold-MIC changes was included. "…These were calculated by division of the MIC used for the experiments and the actual MIC for each isolate (**S1 Table**). For example, for RIF at 1/20xMIC (MIC_{RIF} used in this study is 0.1 µg/mL), the antibiotic concentration used was 0.005. For the ITM 063846, its actual MIC_{RIF} is 0.025. When 0.005 was divided by 0.025, the result showed the fold decrease (1/5=0.02 fold)."

Change 1.2. Drug concentrations used in the experiments and the corresponding ones at different fold-MIC changes were included in the Table S2, which is updated and uploaded as "Revised_S1 Table".

Reviewer #2: While the RIF+AMX/CLV and RIF+CLA+AMX/CLV combinations showed also the strongest effect in luminescence, optical density, 16S rRNA RT-qPCR assay, no correlation was observed with the IS2404 qPCR readout. The statements 'In summary, an overall good correlation was observed between CFU counting and the other methodologies (line 119 and 120), 'in fact, all provided comparable results' (line 283), and 'close correlation between CFU counting and the other methodologies' (line 292) should be modified accordingly. In this context, it should also be discussed more clearly, from which readouts one expects to see a decline associated with the killing of the mycobacteria. Time-kill kinetic data confirmed the high activity of RIF+AMX/CLV and RIF+CLA+AMX/CLV combinations, but the authors should also mention that RIF+CLA yielded comparable results for two isolates (CO5143 and 063846). The exceptional properties of strain 070290 should be commented and it should be made clearer, why isolate CO5143 is also labelled 'exceptional' (line 251). Minor point: in line 252, CO15143 should read CO5143. Figures are of sufficient quality, but minor modifications are required in the description of the results.

Changes 2.1. Modifications regarding the results and discussion of the readouts were included.

*Page 2, Lines 36-37. "…*All methodologies except IS*2404* quantitative PCR provided similar results with a good correlation with CFU counting."

Page 11, Lines 219-223. "…On the contrary, IS*2404* copy numbers remained stable in all conditions, which confirmed that no differentiation could be made between viable and nonviable bacteria with this methodology (**Fig 1E**). Therefore, correlation factors for IS*2404* qPCR were not included in **Table 1**." This would also contribute to explain the expectation of this readout for not to show a decline in the killing of the mycobacteria (*see Comment 1 below*).

Page 13, Lines 228-229. No changes were made since correlation factors for IS*2404* qPCR were not included. "In summary, an overall good correlation was observed between CFU counting and the other methodologies (**Table 1**)."

Page 15, Lines 292-294. "…all of those that were able to differentiate viability provided comparable results, which depicted the availability of a diverse range of biomarkers in BU research."

Page 16, Lines 301-303. "…The close correlation between CFU enumeration and the other methodologies showed that all but IS*2404* qPCR were reliable surrogate markers for detecting *M. ulcerans* live cells."

Comment 1. Discussion on which readouts one expects to see a decline associated with the killing of the mycobacteria were included in *Page 6 (Lines 89-96)*, *Page 11 (Lines 219-223)*, *Page 16 (Lines 307-312)*.

Changes 2.2. Modifications regarding the drug responses of different isolates were included.

Page 14, Lines 258-271. "…At these concentrations, RIF+CLA combination, which is the current treatment for BU, was only active against the ITM 063846 isolate. Interestingly, even the highest RIF concentration tested (100xMIC, MIC increase of 200-fold) against the ITM C05143 isolate was not able to prevent bacterial re-growth, which was not observed when treated at 1xMIC in combination therapy with either of the RIF-containing combinations (**Fig 2, S1 Fig**). Regarding the ITM 070290 isolate, a RIF concentration of 1 µg/mL (10xMIC, MIC increase of 2-fold) was bactericidal (**S1 Fig**). However, a clear trend of interaction could not be identified, which might be due to effective concentrations of the antibiotics being much lower than their actual MIC values, i.e., RIF was tested at concentrations 5- and 20-fold lower than their actual MIC values, while this being 4- and 16-fold reduction for CLA tested at 1xMIC and 1/4xMIC values, respectively. In the case of AMX/CLV, no antibacterial activity was observed for this

isolate even at the highest concentration, which correlated with a MIC increase of 800-fold."

Reviewer #3: yes there is reasonable evidence to support the main claims made in this manuscript. There is a sentence in the results that didn't make sense to me and needs clarification. Lines 248 and 249A generalised trend of interaction was identified for pair-wise RIF+AMX/CLV and triple RIF+CLA+AMX/CLV combinations, which were highly bactericidal, whereas drugs alone had no effect at those concentrations.
Do the authors mean SINGLE drugs alone? If so which specific single drugs? Is this an argument to show that combinations are more potent than single drugs?
Answer: Yes, this sentence refers to single drugs alone and to show that RIF+AMX/CLV and tripe RIF+CLA+AMX/CLV combinations were more potent that these single drugs alone, which are RIF, CLA and AMX/CLV.
Change 3.1: *Page 13, Lines 237-238*. "…The activity of the single compounds alone was first tested in dose response time-kill kinetic studies (**S1 Fig**)."
Change 3.2: *Page 14, Lines 255-256*. "…whereas single drugs alone (RIF, CLA and AMX/CLV) had no effect at those concentrations."

Conclusions

Reviewer #1: The conclusions are broadly supported and the limitations acknowledged. The potential usefulness of the luminescence assay in a clinical setting may be a little exaggerated as although it has advantages over the other methods, it still requires Biosafety level 3 facilities and specialized equipment and reagents that may not always be readily available. It is worrying that the responses of the different strains to the drug combinations used are quite variable. The impact this may have on clinical use should be discussed in detail.
Change 1.3. Clarification on the usefulness of the luminescence assay in laboratories for *in vitro* studies but not in a clinical setting was included.
Pages 16-17, Lines 320-327. "…The turnaround time (TAT) for the 16S rRNA RT/IS*2404* qPCR assay is contingent on the source material, being faster when processing clinical samples than liquid cultures. De facto, this methodology is the most suitable to be used with clinical samples. In a laboratory setting, the TAT for either methodology, OD_{600}, RLU or 16S rRNA RT/IS*2404* qPCR, would be much shorter, with

immediate results compared to the three months needed for the gold-standard methodology (CFU enumeration). More specifically, OD_{600}, and RLU measurements require less manipulation and time that 16S rRNA RT/IS*2404* qPCR."

Pages 17, Lines 339-341. "...In fact, based on cost-effective considerations including time to results, sample processing and manipulation time, this was the methodology selected to expand the study to further clinical isolates."

Change 1.4. *Pages 18. Lines 352-361.* "…In general, specific responses to drugs alone and in combination for each isolate were variable in our assays. This is not unexpected when considering the intrinsic variability in MIC distributions among *M. ulcerans* clinical isolates (32). In the case of these bacteria, there are not established epidemiological cut-off (ECOFF) values and and clinical breakpoints according to international antimicrobial susceptibility testing guidelines (33,34). However, antibiotic concentrations used in this study were much lower than the ones reported in plasma in several pharmacokinetic studies (35–39). Furthermore, this variability does not seem to play a significant role when translating to clinical efficacy considering that the efficacy of the standard treatment for BU (RIF+CLA for eight weeks) is as high as 96% (18)."

Reviewer #2: (No Response)

Reviewer #3: The conclusions are supported by the data presented. I would like to see some more information about laboratory turn around time (potentially) if the faster methods described in this research were to be implemented.

Change 3.3. *Pages 16-17, Lines 320-327.* "…The turnaround time (TAT) for the 16S rRNA RT/IS*2404* qPCR assay is contingent on the source material, being faster when processing clinical samples than liquid cultures. De facto, this methodology is the most suitable to be used with clinical samples. In a laboratory setting, the TAT for either methodology, OD_{600}, RLU or 16S rRNA RT/IS*2404* qPCR, would be much shorter, with immediate results compared to the three months needed for the gold-standard methodology (CFU enumeration). More specifically, OD_{600}, and RLU measurements require less manipulation and time that 16S rRNA RT/IS*2404* qPCR."

Editorial and Data Presentation Modifications

Reviewer #1: It would be very helpful if the assay MICs and the actual MICs could be distinguished more clearly in the text and figures.

Answer. Text was rewritten and more clarification on MICs was included (*see Changes 1.1, 1.2 and 2.2.*).

Reviewer #2: (No Response)

Reviewer #3: (No Response)

Summary and General Comments

Reviewer #1: The combined use of amoxicillin/clavulanate with rifampacin/clarithromycin for the treatment of Buruli Ulcer is currently undergoing clinical trials. This paper is an in vitro study that compares several different methodologies for following the effect of the drugs individually and in combination on M.ulcerans concentrations. The authors report that a luminescence based assay is the most cost-effective method that correlates well with the gold standard CFU counting and go on to compare the results obtained with this assay using a panel of bacterial strains. The work is well done and adds an alternative method to those currently routinely used to monitor M. ulcerans growth. The writing is generally good but sometimes lacks clarity.

Reviewer #2: Overall, the manuscript provides valuable information for the choice of the different types of measurement for time-kill assays, dependent on the purpose of a study. In particular, the discussion on the degradation kinetics of antibiotics may animate discussions on study design.

Reviewer #3: This is a technical study that is potentially useful. My skill set is predominantly clinical infectious diseases including the management of Buruli patients, hence I suggest also a review by a microbiology scientist or medical microbiologist. Nevertheless it is interesting and convincing to see this in vitro demonstration of the effectiveness at a microbiological level of the combinations of rifampicin plus amox/clav. I think it is unlikely that we will ever use triple drug therapy in clinical practice as rifampicin and clarithromycin are already highly effective. However there are times we

need to replace one or other of these drugs for patients who develop antibiotic intolerance. It is good to know that there is consistent laboratory support at least for Rifampicin with another safe and licensed antibiotic.

Comments. We appreciate reviewers' comments.
